# Supplementary material for: Critical Assessment of Whole Genome and Viral Enrichment Shotgun Metagenome on the Characterization of Stool Total Virome in Hepatocellular Carcinoma Patients
Source: Viruses. 2022 Dec 24;15(1):53. doi: 10.3390/v15010053 (PMC9866815; doi:10.3390/v15010053)
Supplement: Supplementary file 1 [file viruses-15-00053-s001.zip › Supplementary_Material.pdf]

Supplementary Material

Figure S1. (a) The schematic representation of this study. Faecal samples of 6 HCC patients and 6 healthy controls were collected and then sequenced using WGSM and VLPM, respectively. (b) Study design. (c) Evaluation of sequencing depth. Genetic contents flow was depicted.

Figure S2. The rarefaction curves of the samples given (a) contig length >= 3kb, (b) contig length >= 5kb and (c) contig length >= 8kb. The coverage rate remains >= 75%. (d) The alpha diversities (Shannon index) given contig length >= 10kb and coverage rate >= 75%. The alpha diversities (observed features) given (e) contig length >= 3kb, (f) contig length >= 5kb and (g) contig length >= 8kb. The coverage rate remains >= 75%.

Figure S3. The rarefaction curves of the samples given (a) coverage rate remains >= 10%, (b) coverage rate remains >= 25%, (c) coverage rate remains >= 50% and (d) coverage rate remains >= 90%. The contig length remains >= 10kb. The alpha diversities (observed features) given (e) cov-erage rate remains >= 10%, (f) coverage rate remains >= 25%, (g) coverage rate remains >= 50% and (h) coverage rate remains >= 90%. The contig length remains >= 10kb. (i) The ratio of reads mapped to virus contigs to those mapped to all the assembled contigs.

Figure S4. The principal coordinates analysis plots based on (a) the WGSM samples, (b) the VLPM samples, (c) the healthy controls, (d) the HCC patients and (e) the combination of all the samples, respectively. The plots used Jaccard dissimilarities. (f) Virus compositions at the family level.

Table S1. QC table of the sequencing data. The number of reads from raw data and those after deduplicates, host contamination removal, low-quality reads and removal of rRNA reads.

Table S2. The STORMS checklist.

Table S3. Enriched viral signatures from MaAsLin2. Abundance of contigs with same annota-tions were merged into one representative record.

Table S4. The FPKM of the detected RNA Viruses.
