# Supplementary figures and images for: Critical Assessment of Whole Genome and Viral Enrichment Shotgun Metagenome on the Characterization of Stool Total Virome in Hepatocellular Carcinoma Patients
Source: Viruses. 2022 Dec 24;15(1):53. doi: 10.3390/v15010053 (PMC9866815; doi:10.3390/v15010053)

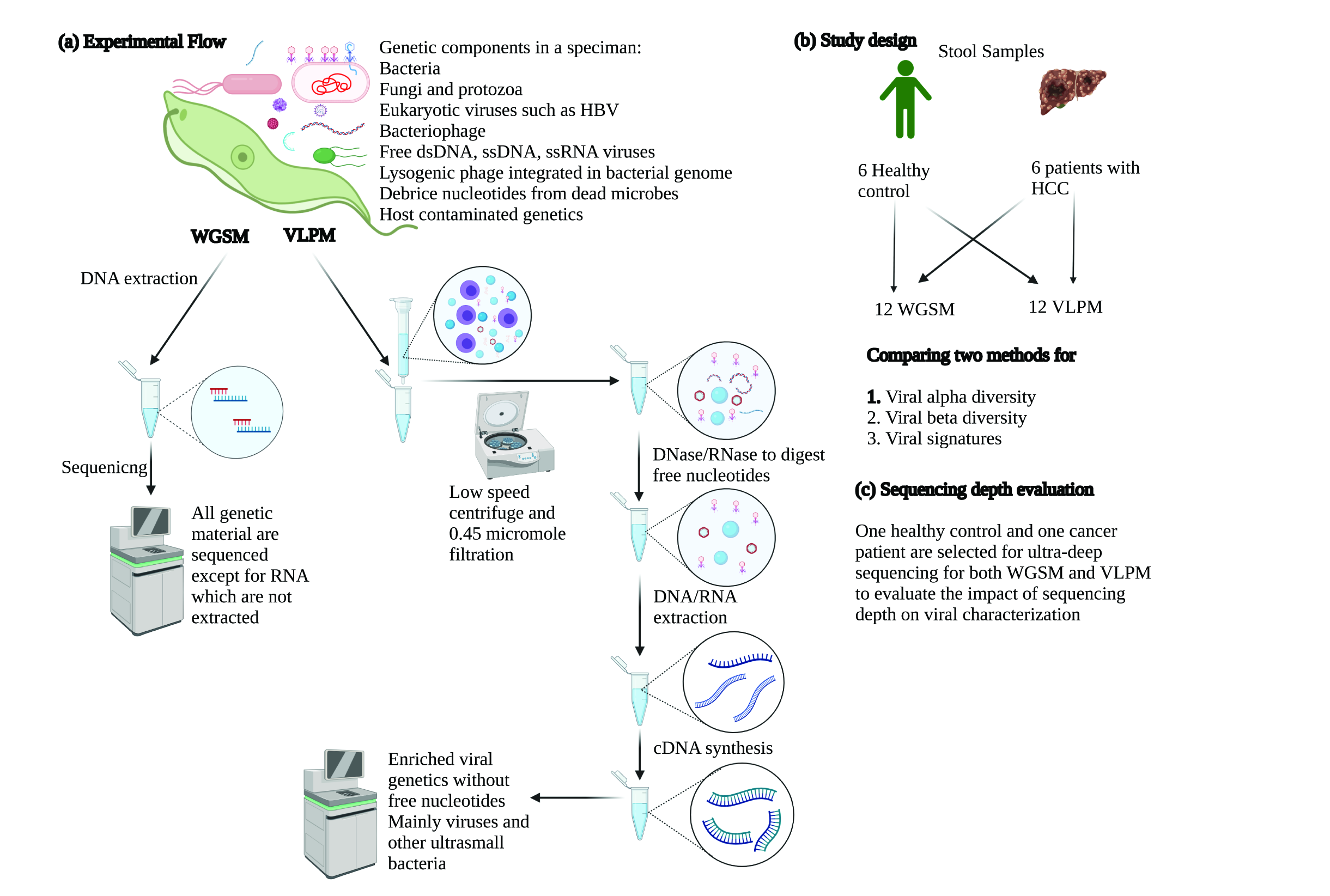

Supplement: Supplementary file 1 [file viruses-15-00053-s001.zip › FigS1.tif]

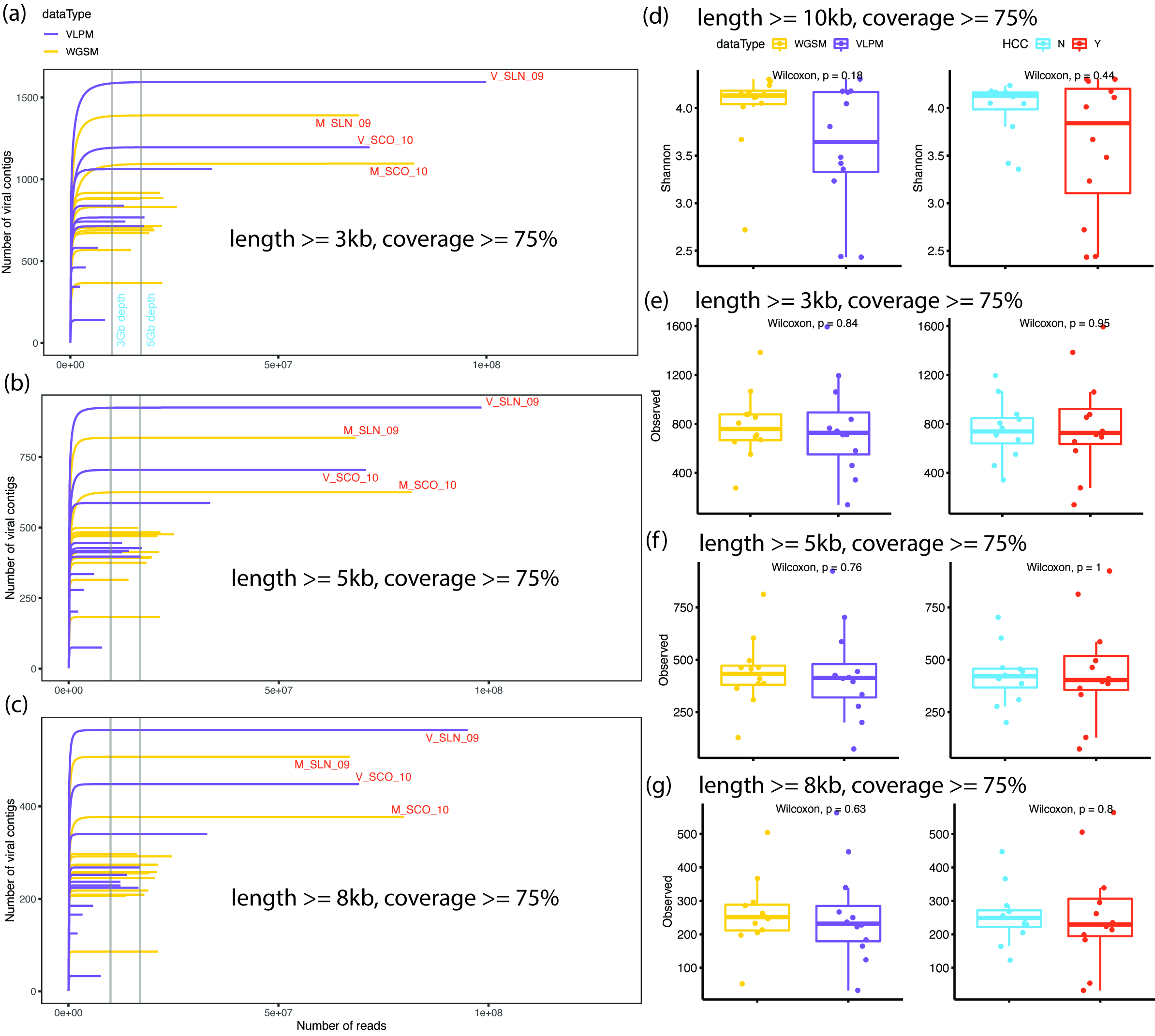

Supplement: Supplementary file 1 [file viruses-15-00053-s001.zip › FigS2.tif]

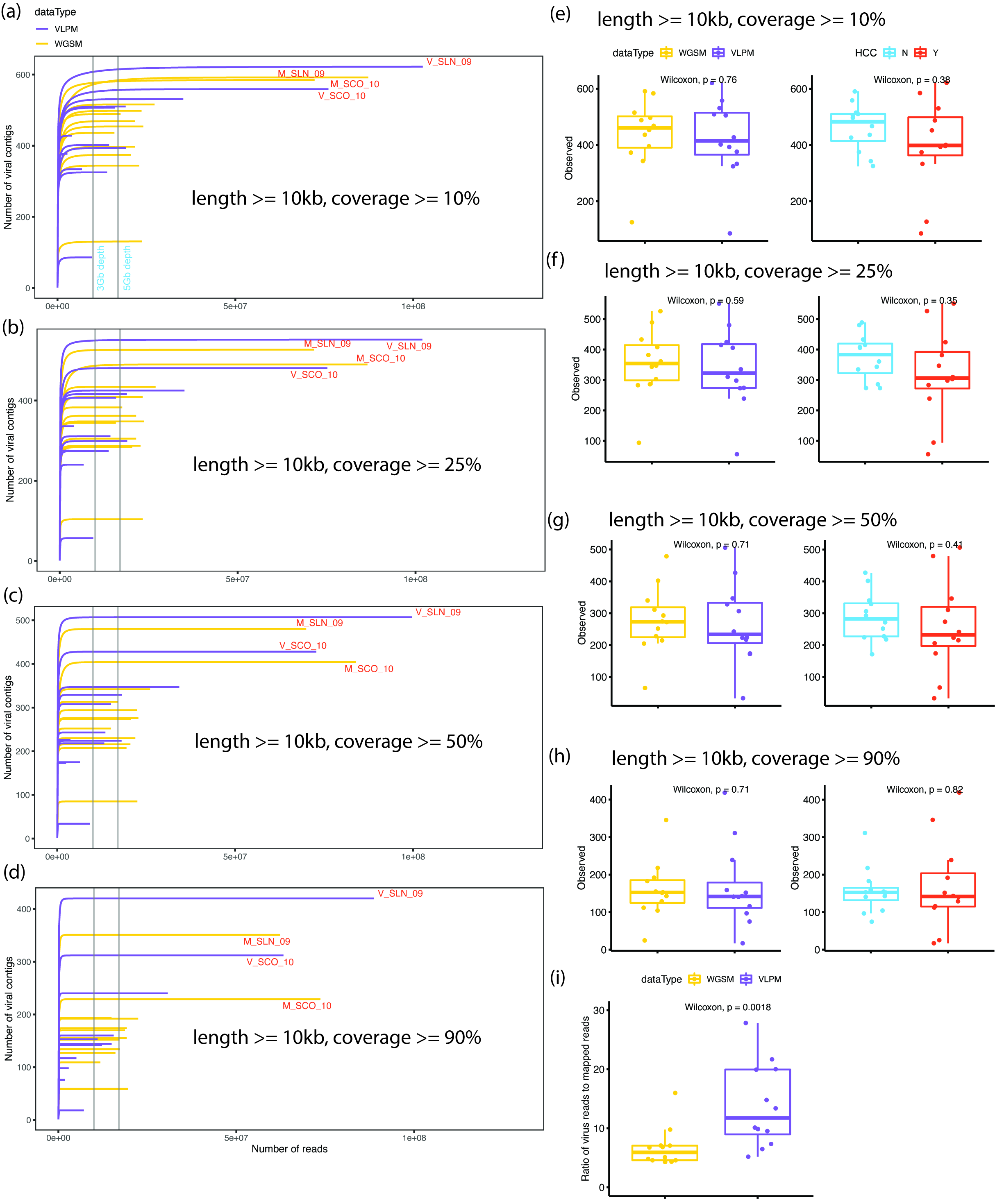

Supplement: Supplementary file 1 [file viruses-15-00053-s001.zip › FigS3.tif]

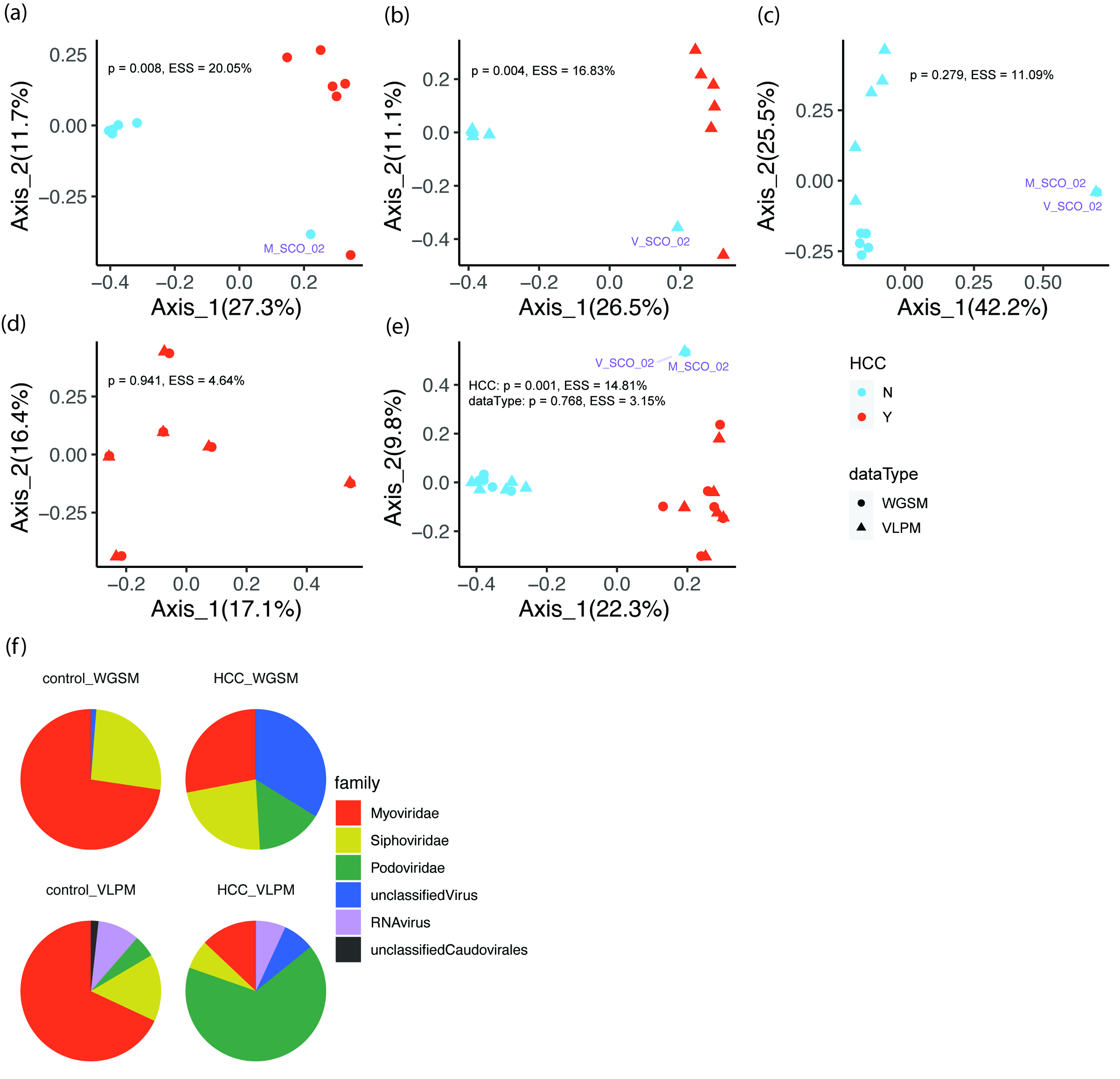

Supplement: Supplementary file 1 [file viruses-15-00053-s001.zip › FigS4.tif]
